# Case Report: Complete remission of refractory Langerhans cell sarcoma following CLAG-M chemotherapy and allogeneic hematopoietic stem cell transplant

**DOI:** 10.3389/fonc.2026.1714214

**Published:** 2026-03-04

**Authors:** Aubree Mades, Lydia Chow, Alireza Ghezavati, Amir Ali, Kimberly Schiff, Lakshmi Savitala-Damerla, Imran Siddiqi, Brandon Tang, George Yaghmour

**Affiliations:** 1 Jane Anne Nohl Division of Hematology and Center for the Study of Blood Diseases, Keck School of Medicine of University of Southern California, Los Angeles, CA, United States; 2 Department of Pathology, Keck School of Medicine of University of Southern California, Los Angeles, CA, United States

**Keywords:** allogeneic transplantation, case report, immunotherapy, Langerhans cell histiocytosis, Langerhans cell sarcoma, MAPK pathway

## Abstract

Langerhans Cell Sarcoma (LCS) is an extremely rare and aggressive neoplasm with limited consensus on optimal treatment. We report the case of a 56-year-old woman with refractory Langerhans Cell Histiocytosis (LCH) that transformed into Langerhans Cell Sarcoma (LCS) who achieved complete remission following chemotherapy with cladribine, high-dose cytarabine, G-CSF, and mitoxantrone (CLAG-M), and subsequent allogeneic hematopoietic stem cell transplantation (HSCT). This case highlights the potential role of allogeneic HSCT as an effective therapeutic option for refractory LCS and underscores the importance of reporting additional cases to guide future management strategies in this rare malignancy.

## Introduction

Langerhans cell sarcoma (LCS) is a rare and aggressive malignancy originating from pathologic Langerhans cells. Its diagnosis is often difficult, and initial histologic findings may lead to misdiagnosis, as reported in prior cases such as when Charfi et al. described a patient initially diagnosed with Hodgkin’s lymphoma who was later found to have LCS after disease recurrence (1). These tumors often exhibit resistance to conventional therapies and carry poor prognosis. LCS may develop *de novo* or through transformation from Langerhans Cell Histiocytosis (LCH), which is characterized by CD1a+/Langerin+ dendritic cells. Because of the limited number of reported cases and absence of standardized treatment protocols, management strategies vary and are frequently extrapolated from other hematologic malignancies. Recent literature highlights that Langerhans cell sarcoma is a clinically and prognostically heterogeneous disease. In a recent systematic review of 88 reported cases, Dezzani et al. demonstrated that outcomes vary widely and are influenced by both disease extent and association with other hematologic malignancies. Patients with multisystem involvement had worse survival compared with those with single-system disease, supporting the importance of subclassifying LCS based on disease spread. The review also emphasized that LCS frequently shows focal or heterogeneous CD207 (Langerin) expression, reinforcing the need to explicitly report Langerin status when describing tumor phenotype (2). The Charfi case, in which chemotherapy with etoposide, methylprednisolone, cytarabine, and cisplatin (ESHAP) achieved partial disease control but ultimately failed, underscores the need for more effective treatment approaches (1). Here, we present a rare case of refractory LCS treated with CLAG-M chemotherapy followed by allogeneic HSCT, achieving remission.

## Case description

**Figure 1:**
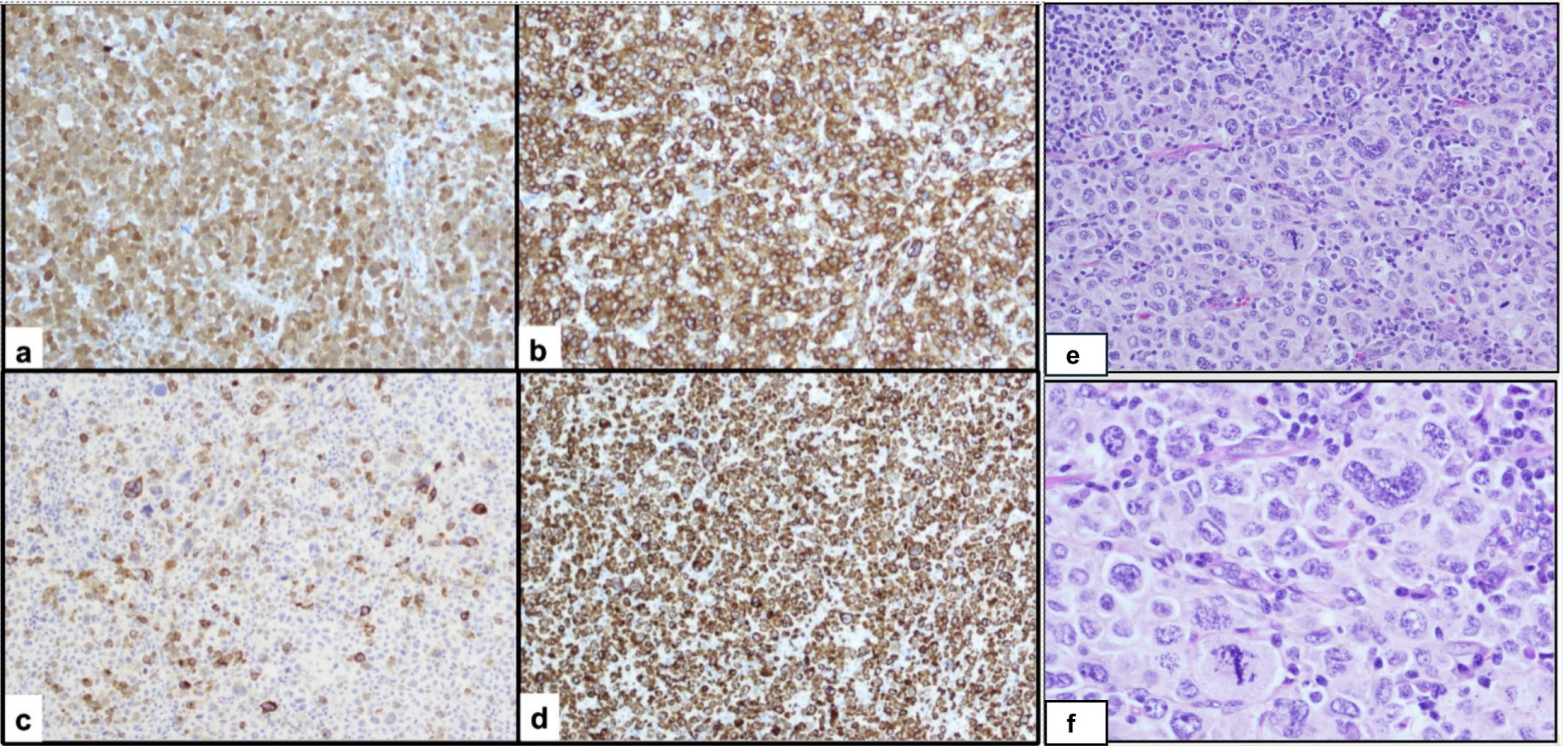
Histopathologic and immunophenotypic features of Langerhans cell sarcoma. **(E)** Hematoxylin and eosin–stained section (20×) demonstrating sheets of large, highly pleomorphic tumor cells with overtly malignant cytologic features, including irregular to convoluted and occasionally bizarre nuclei, coarse chromatin, prominent nucleoli, abundant cytoplasm, and increased mitotic activity, including atypical forms. **(F)** Higher magnification (40×) highlighting marked cytologic atypia and atypical mitotic figures. Immunohistochemical staining shows tumor cell positivity for S100 **(A)**, CD1a **(B)**, and CD207/Langerin with focal expression **(C)**, confirming Langerhans cell lineage. Tumor cells also demonstrate aberrant expression of T-cell markers, including diffuse CD3 positivity **(D)**. Additional T-cell markers (CD2, CD4, CD5) were variably positive (not shown). The Ki-67 proliferation index was markedly elevated (~80%).

**Figure 2:**
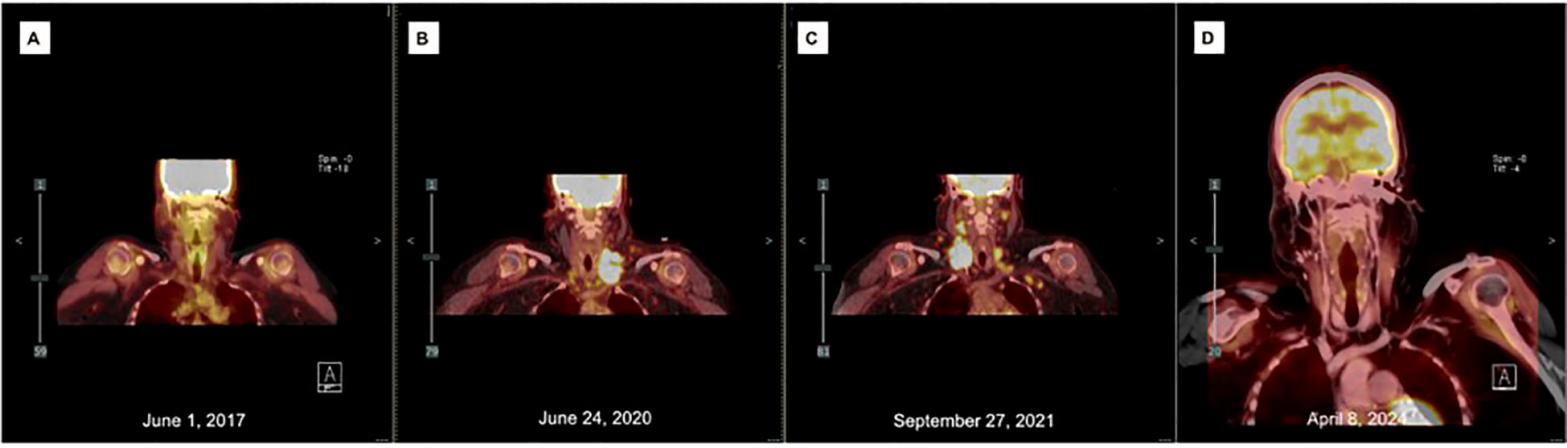
PET scans of the neck throughout treatment course: **(A)** At diagnosis, pathology consistent with Langerhans Histiocytosis, left supraclavicular LN measuring 1.3cm with SUV 2.2; **(B)** Transformation to Langerhans Sarcoma, left supraclavicular nodal mass measuring 4.5cm x 2.7cm with SUV 18.6; **(C)** Following six cycles of cladribine and seven cycles Pembrolizumab, right supraclavicular nodal mass measures 3.6cm x 2.8cm with SUV 16.1; **(D)** 12 months following allogeneic matched unrelated donor transplant, most prominent LN measures 6mm with SUV 2.3.

A 56-year-old Hispanic woman with no past medical history was diagnosed with Langerhans Cell Histiocytosis (LCH) in 2017 after presenting with left supraclavicular lymphadenopathy (LAD) and rash. An excisional lymph node biopsy (LN) revealed atypical Langerhans cell proliferation (CD1a+, S100+, Langerin+) and was negative for BRAF V600E mutation (**Figure 1**). Positron emission tomography/computed tomography (PET/CT) demonstrated a 1.3cm left supraclavicular LN with a standardized uptake value (SUV) of 2.2, a mildly enlarged left axillary LN, and tiny lucencies in the lower cervical/upper thoracic spine and iliac bones (**Figure 2**). Magnetic resonance imaging (MRI) showed no pathologic bone lesions. Skin biopsy was consistent with Langerhans neoplasm with Ki67 90% and 60% PDL1 expression by IHC, with no clinically significant mutations on next-generation sequencing. Although the high Ki-67 proliferation index seen in the 2017 skin biopsy was unusual for adult Langerhans Cell Histiocytosis, the initial diagnosis of LCH was considered appropriate at the time based on the overall clinical and pathologic findings. These included limited disease involvement, skin-predominant presentation, and an immunophenotype consistent with Langerhans lineage (CD1a^+^, S100^+^, Langerin^+^), without clear malignant cytologic features or aggressive systemic disease. In retrospect, given the patient’s later progression with increasing nodal involvement, marked cytologic atypia, and aggressive clinical behavior, the possibility that an early Langerhans Cell Sarcoma was present at initial diagnosis cannot be excluded. However, a definitive diagnosis of LCS was only established on subsequent biopsies demonstrating overt malignant features and disease progression. Given limited involvement, she was initiated on topical steroids and a prednisone taper, then placed on oral methotrexate 60mg weekly for three years.

In June 2020, PET-CT demonstrated growth of the left supraclavicular LN to 4.5 cm with SUV 18.6, new cervical chain involvement, and development of supraclavicular lymphadenopathy (**Figure 2**). Lymph node biopsy revealed transformation to Langerhans cell sarcoma (LCS) (CD4^+^, CD1a^+^, S100^+^) (**Figure 1**). Immunophenotyping of the LCS lesion demonstrated expression of CD1a and S100, with focal CD207 (Langerin) positivity, confirming Langerhans cell lineage differentiation. Tumor cells showed aberrant co-expression of T-cell markers, including diffuse CD3 positivity and variable expression of CD2, CD4, and CD5, while CD7 was negative. To formally exclude T-lymphoblastic lymphoma/leukemia, additional markers were assessed and were negative for TdT and CD34. The overall phenotype of CD1a+, S100+, CD207/Langerin (focal), high proliferative index, overt cytologic atypia, and aberrant but non–lineage-defining T-cell marker expression was diagnostic of Langerhans Cell Sarcoma. The patient underwent six cycles of cladribine 5mg/m2 D1-D5.

Following cladribine treatment, PET-CT demonstrated interval worsening of prior LAD and new left periauricular LAD, confirmed as refractory LCS on biopsy. She was treated with 7 cycles of pembrolizumab. Although her biopsy showed 60% PD-L1 expression, PD-L1 is not a validated biomarker in histiocytoses and published responses to immunotherapy in this setting are variable. Subsequent PET-CT showed disease progression.

At this time, the patient transferred her care to our institution. She underwent bilateral modified radical neck dissection, which revealed extensive tumor involvement of the bilateral neck and prevertebral muscles extending into the mediastinum. Over 50 lymph nodes were involved, with histopathological findings consistent with Langerhans Cell Sarcoma. Molecular analysis demonstrated absence of the BRAF V600E mutation and identified a pathogenic MAP2K1 (MEK1) mutation, indicating activation of the MAPK pathway. Notably, there was aberrant co-expression of multiple T-cell markers, with a particularly diffuse CD3 expression. Prior to initiation of CLAG-M chemotherapy, laboratory evaluation on December 27, 2021 demonstrated a white blood cell count of 7.51 ×10³/µL with a normal differential, including monocytes at 5.2%, hemoglobin of 10.7 g/dL, and platelet count of 145 ×10³/µL. Bone marrow biopsy (BMB) showed 40% cellular marrow with no abnormal immunophenotypic populations and normal cytogenetics. PET-CT showed new right adrenal gland thickening and mediastinal LAD.

Given intolerance to MEK inhibitors due to gastrointestinal toxicity, the patient was initiated on induction therapy with one cycle of CLAG-M in December 2021, followed by consolidation cycles with CLAG in August 2022. Following cycle two, PET-CT showed increased size and avidity of multiple retroperitoneal lymph nodes. A repeat PET-CT before the transplant demonstrated decreased but persistent thoracic and abdominopelvic LNs.

In April 2023, the patient underwent an allogeneic stem cell transplant using mismatched unrelated donor mMUD 8/10. She received a myeloablative, radiation-based conditioning regimen, consisting of fludarabine from D-6 to D-4, followed by total body irradiation to a total dose of 1200 cGy administered twice daily from D-3 to D-1. Post-transplant prophylaxis against graft-versus-host disease (GVHD) included cyclophosphamide/mesna on day +3 and day +4, followed by tacrolimus and mycophenolate. She achieved complete donor engraftment. Her only GVHD complication was Grade 2 skin acute GVHD, which resolved with prednisone. Post-transplant PET-CTs have shown complete resolution of the previous LAD (**Figure 2**). Over two years post-transplant, the patient continues showing no signs of LCS recurrence and has maintained Graft-versus-Host Disease-Free, Relapse-Free two-year Survival.

The overall clinical course, including diagnosis, treatments, and outcomes, is summarized in **Table 1**.

**Table 1:** Timeline of diagnosis, treatments, and outcomes in a patient with refractory Langerhans cell sarcoma.

Date/Time	Event & Intervention	Outcome
2017	Initial diagnosis: LCH (CD1a+, S100+, Langerin+, BRAF V600E−). Treated with topical steroids → prednisone taper → methotrexate 60 mg weekly × 3 years.	Stable disease while on therapy
June 2020	Transformation to LCS. PET-CT: 4.5 cm LN (SUV 18.6). LN biopsy: LCS (CD4+, CD1a+, S100+).	Began cladribine × 6 cycles
Late 2020	Refractory disease on cladribine.	Pembrolizumab × 7 cycles, but PET-CT showed progression
2021	Progression: Bilateral neck dissection → extensive LCS; MAP2K1 mutation; CD3 aberrant expression.	Further progression
Dec 2021	Induction: CLAG-M (cycle 1).	Partial response
Aug 2022	Consolidation: CLAG (cycle 2).	Residual LAD, mixed response
Apr 2023	Allogeneic HSCT (mMUD 8/10) with myeloablative, radiation-based conditioning + PTCy prophylaxis	Engraftment achieved; grade 2 skin GVHD resolved; PET-CT showed complete remission
2023–2025	Serial follow-up with PET-CTs.	Ongoing complete remission > 2 years

## Discussion

Histiocytic disorders are now classified using the revised Histiocyte Society system as well as the updated WHO 5th Edition Hematolymphoid Tumors classification and the International Consensus Classification (ICC) (3, 4). Within these frameworks, Langerhans Cell Histiocytosis (LCH) belongs to the L-group histiocytoses, whereas Langerhans Cell Sarcoma (LCS) is categorized as a malignant histiocytosis, reflecting its marked cytologic atypia, high proliferative index, and aggressive clinical course. L-group histiocytoses include LCH and related dendritic cell disorders, while malignant histiocytoses include LCS and histiocytic sarcoma as defined by Emile et al. and Bigenwald et al. (5, 6). LCS demonstrates capacity for systemic dissemination and carries a significantly worse prognosis compared with LCH. According to both the WHO 5th Edition and the ICC, LCS is formally classified as a malignant histiocytic neoplasm characterized by marked cytologic atypia and high proliferative activity.

Langerhans Cell Sarcoma (LCS), while sharing immunophenotypic markers with LCH (CD1a+, Langerin+), is distinguished by marked cellular atypia and prominent mitotic activity. These are features required for diagnosis (7). LCS is a rare and highly aggressive malignancy (6). It may arise *de novo* or from pre-existing LCH, exhibiting aggressive clinical behavior, malignant histopathologic features, and capacity for widespread systemic dissemination. A 2015 systematic review of 66 LCS cases revealed a median age at presentation of 50 years, with most patients over age 20, and a dismal prognosis. The 1-year disease-specific survival (DSS) was 58% and 5-year DSS was 28% (7). None of the patients with disseminated disease survived beyond 5 years. Treatment strategies vary due to rarity, including surgical resection for localized disease and chemotherapy with cyclophosphamide, doxorubicin, vincristine, and prednisone (CHOP), as well as platinum-based regimens and ifosfamide.

The main differential diagnosis of Langerhans Cell Sarcoma includes indeterminate cell histiocytosis and other dendritic cell neoplasms with overlapping immunophenotypic features. Both LCS and indeterminate cell histiocytosis can demonstrate CD1a expression and cytologic atypia, which can make distinction challenging. However, LCS is characterized by overt malignant cytologic features, high proliferative activity, and retention of CD207 (Langerin) expression, whereas indeterminate cell histiocytosis lacks Langerin expression and generally follows a more indolent clinical course. Aberrant expression of T-cell markers has been reported in LCS and should prompt exclusion of T-lymphoblastic lymphoma/leukemia using markers such as TdT and CD34 (5, 8). Although our patient demonstrated PD-L1 expression, PD-L1 is not a validated prognostic or predictive biomarker in histiocytic neoplasms, and reported responses to pembrolizumab in LCS and other histiocytoses have been inconsistent.

In our patient, who demonstrated a MAP2K1 mutation and could not tolerate MEK inhibitors due to GI toxicity, cladribine-based therapy was initiated, followed by CLAG-M, and ultimately allogeneic HSCT from a mismatched unrelated donor (MUD) using myeloablative, radiation-based conditioning and post-transplant cyclophosphamide (PTCy). This resulted in complete remission exceeding two years. This outcome reinforces the potential role of allogeneic HSCT as a curative option in relapsed or refractory LCS. Continuous surveillance with PET-CT, marrow biopsies, and CNS imaging remains essential. Preemptive post-transplant MEK inhibition or graft-versus-tumor strategies may offer future therapeutic refinements.

### Patient perspective

The patient expressed gratitude for the care received and the outcome achieved. She was appreciative of the multidisciplinary approach and felt well supported throughout her treatment and recovery. She continues to engage in regular follow-up with her care team.

## Conclusion

Our case demonstrates that allogeneic HSCT can achieve remission in refractory Langerhans Cell Sarcoma, highlighting its potential as a curative strategy in this rare malignancy. Exploration of post-transplant targeted therapies may further optimize long-term outcomes.

## Data Availability

The original contributions presented in the study are included in the article/supplementary material. Further inquiries can be directed to the corresponding author.
